# An Electrochemical Evaluation of Novel Ferrocene Derivatives for Glutamate and Liver Biomarker Biosensing

**DOI:** 10.3390/bios11080254

**Published:** 2021-07-28

**Authors:** Geok Hong Soon, Mary Deasy, Eithne Dempsey

**Affiliations:** 1 Centre of Applied Science for Health (CASH), Technological University of Dublin, Tallaght, D24 FKT9 Dublin, Ireland; geokhong.soon@gmail.com (G.H.S.); Mary.Deasy@TUDublin.ie (M.D.); 2 Department of Chemistry, Kathleen Lonsdale Institute for Human Health, Maynooth University, Maynooth, W23 F2H6 Co. Kildare, Ireland

**Keywords:** glutamate biosensor, liver biomarker, *γ*-glutamyl transpeptidase, electrochemical sensing, ferrocenes, polymer films

## Abstract

Here, we present an evaluation of two new monosubstituted ferrocene (Fc) derivatives, 3-(1H-pyrrol-1-yl)propanamidoferrocene and 1-hydroxy-2-[2-(thiophen-3-yl)-ethylamino]ethylferrocene, as glutamate oxidase mediators, together with their preparation and characterisation. Taking into consideration the influence of the electronic effects of substituents on the redox potentials of the Fc species, two candidates with pyrrole or thiophene moieties were proposed for investigation. Film studies involved potential sweeping in the presence of pyrrole or 3,4-ethylenedioxythiophene monomers resulting in stable electroactive films with % signal loss upon cycling ranging from 1 to 7.82% and surface coverage (Γ) 0.47–1.15 × 10^−9^ mol/cm^2^ for films formed under optimal conditions. Construction of a glutamate oxidase modified electrode resulted in second-generation biosensing with the aid of both cyclic voltammetry and hydrodynamic amperometry, resulting in glutamate sensitivity of 0.86–1.28 μA/mM and K_m_ _(app)_ values over the range 3.67–5.01 mM. A follow-up enzyme assay for liver biomarker *γ*-glutamyl transpeptidase realised unmediated and mediated measurement establishing reaction and incubation time investigations and a realising response over <100 U/L *γ*-glutamyl transpeptidase with a sensitivity of 5 nA/UL^−1^.

## 1. Introduction

Metallocenes are a class of sandwich compounds in which one of the *d*-block transition metals is bound between two parallel *η*^5^- or *π*-bonding aromatic cyclopentadienyl (Cp) rings. In the case of ferrocene (Fc), the first known archetypal metallocene, this metal is iron. In terms of electrochemistry in particular, Fc derivatives have made frequent appearances as redox mediators in numerous literature reviews and reports, as they have been shown to facilitate rapid electron transfer between enzymes and electrodes. With the combination of efficiency, stability in reduced form, pH-independent redox potentials, ease of synthesis and versatility in substitution, Fc has exceeded other organometallic compounds in its applications in biosensors. However, with its chemical structure of two *π*-bonding Cp rings, intensive efforts have been made to modify one or both of the *π*-bonding Cp rings to render Fc more hydrophilic or otherwise more suitable to be employed as a redox mediator [1,2].

Glutamate is a key neurotransmitter in the central nervous system and is involved in all aspects of normal brain functioning [3]. Disrupted glutamate homeostasis has been proposed to be related to various neurological conditions, e.g., schizophrenia, epilepsy, Parkinson’s disease and Alzheimer’s disease [4]. In relation to glutamate biosensing, the design and applications of electrochemical glutamate biosensors have been the subject of a recent review [5]. Huang et al. [6] reported the use of Fc-functionalised, single-walled carbon nanotubes SWCNT, non-covalent, nano-hybrid, inter-digitised construction films for l-glutamate detection. Immobilisation of Fc onto (SWCNT) surface was carried out by re-dispersing the mixture which was first mixed and shaken at room temperature overnight in ethanol using sonication. The modified electrode was fabricated by welding a platinum wire onto glass slides which were modified with Fc/SWCNT layers. The optimal thickness of the Fc/SWCNT was determined using scanning electron microscopy (SEM) experiments (side-on). The electrode was then partly mounted with epoxy resin, and a small area of the film was left exposed. With excellent stability in aqueous and organic media such as ethanol and acetone, the SWCNT/Fc electrode exhibited high catalytic efficiency, high sensitivity and fast response during detection of low l-glutamate concentration of 1 µM. A good linear relationship between current and concentration was obtained from 1 to 7 μM. A glutamate microbiosensor designed as a tool in neurochemistry was reported based on reduced graphene oxide with a Prussian blue/gold nanoparticle composite with LOD of 41.3 nM over the range 50 nM–40 μM [7]. Amperometric glutamate oxidase (GlutOx) electrodes based on multilayers of polymeric films were reported with films consisting of three layers [8]; the first being the electropolymerised film of 1,3-diaminobenzene; the second consisted of GlutOx entrapped in photopolymerised poly(vinylferrocene)-poly(ethyleneglycol) hydrogel polymer; the final layer was the dialysis membrane. Such a configuration was reported to eliminate interferences. The measurement was made at a potential of 85 mV vs. Ag|AgCl. This flow injection system biosensor responded to l-glutamate between 0.5 and 8.0 mM with a sensitivity of 9.48 nA mM^−1^. The biosensor was stable for 5 days of continuous use (250 assays) and retained 60% activity after 16 days.

Other recent reports include glutamate oxidase immobilised on ZnO nanorods in a polypyrrole film, enabling detection down to 0.18 nm within 5 s [9] and glutamate oxidase immobilisation on polypyrrole nanoparticles at a polyaniline gold electrode [10]. Batra et al. 2013 [11] reported glutamate oxidase on carboxylated multiwalled carbon nanotubes gold nanoparticles/chitosan composite film, while Borisova et al. [3] have monitored glutamate release from brain nerve terminals using an amperometric glutamate biosensor with selectively ensured via the use of the permselective membrane poly(m-phenylenediamine). A glass capillary-based enzyme sensor comprised of an osmium poly(vinylpyridine) redox polymer coated with glutamate oxidase was employed for real-time monitoring of l-glutamate. The substrate of interest was released by high-frequency stimulation in region CA1 of mouse hippocampal slices in combination with the recoding of excitatory postsynaptic potentials (fEPSPs). The l-glutamate level recorded was 15 ± 6 μM (n = 8) and 3 ± 1 μM (n = 5) in the presence and absence of persistent fEPSPs enhancement [12].

Liver disease may be preliminarily detected through routine blood testing, and liver function tests are conducted to evaluate various functions (e.g., metabolism, storage, filtration and excretion). These functions are performed by liver enzymes that are naturally found in liver cells and tend to leak out into the bloodstream when liver cells are injured. Alanine aminotransferase (ALT) and aspartate aminotransferase (AST) are found not only in the liver but also in other body tissues, particularly the heart and muscles. The ratio of ALT to AST is often used in differentiating between causes of liver damage [13]. *Gamma*-glutamyl transpeptidase (GTP) is found in most organs, including the liver, but not muscles. It can be used to differentiate between liver and bone diseases, as both cause elevated ALP. GTP is more susceptible to enzymatic induction by alcohol than AST or ALT, though elevated GTP levels may be caused by liver damage. GTP is passed from the liver into bile and then excreted into urine by the kidneys. GTP elevation can be triggered by numerous conditions such as cholestasis, liver cirrhosis, viral hepatitis, fatty liver, porphyria, toxic liver damage, pancreatitis and pancreatic cancer, myocardial infarction, nephrotic syndrome, diabetes mellitus, right heart failure, obesity, nicotine abuse and brain tumours. Strong association of GTP with carcinoembryonic antigen in colon cancer leads to a metastatic spread to the liver, and hence increase in enzyme levels in neoplastic diseases that are similarly supportive of a diagnosis of hepatic metastases [14]. Isoenzyme-II of GTP is reported to be the best biomarker during preliminary stages of primary liver cell carcinoma.

GTP also plays a major role in cellular glutathione homeostasis and is active in regulating glutathione metabolism. It plays a vital role in protecting and detoxifying at blood–brain barriers, e.g., metabolism of foreign substances via mercapturic acid pathway, and is involved in catalysing transfer of glutamyl residues of different peptides. The normal level of GTP activity in healthy male and female adults is <55 IU/L (international units per litre) and <38 IU/L, respectively (abnormal levels can go up to 500 IU/L).

There has been considerable research on ultraviolet spectrometric detection of GTP with derivatives of glutathione or L-γ-glutamyl-*p*-nitroanilide as substrates [15] but only a few reports on the unmediated electrochemical detection of the enzyme [16,17]. Upadhyay et al. reported on the quantitative determination of GTP activity concerning the use of a miniaturised analysis system using γ-l-glutamyl-l-glutamate and glycyl-glycine as the substrates with immobilised GlutOx on a platinum working electrode surface [16]. GTP catalyses the transfer of γ-glutamyl moiety from γ-glutamyl peptides, e.g., γ-l-glutamyl-l-glutamate to other peptides or amino acids, e.g., glycyl-glycine, resulting in the production of l-glutamate. These enzymatic reaction sequences are presented as follows.

$$\gamma -L-\mathrm{glutamyl}-L-\mathrm{glutamate}\;\overset{\mathrm{GTP}}{\to }\;\gamma -L-\mathrm{glutamyl}-\mathrm{glycyl}-\mathrm{glycine}\;+\mathrm{glycyl}-\mathrm{glycine}\;\;\;\;\;\;+L-\mathrm{glutamate}\;L-\mathrm{glutamate}+O_{2}+H_{2}O\;\overset{\mathrm{GlutOx}}{\to }\;\alpha -\mathrm{ketoglutrate}+{\mathrm{NH}}_{3}+H_{2}O_{2}$$

Of interest is the development of devices such as amperometric biosensors for accurate and rapid detection of liver function biomarkers such as GTP at physiological levels. Presented here is a broad investigation into the electrochemical polymerisation of two Fc derivatives, 3-(1H-pyrrol-1-yl)propanamidoferrocene (**2**) and 1-hydroxy-2-[2-(thiophen-3-yl)-ethylamino]ethylferrocene (**6**), with pyrrole/EDOT in aqueous electrolytic media. The species were incorporated into a polymeric network of polypyrrole or polythiophene for biosensing purposes and the generation of novel redox polymers for electrochemical detection of l-glutamate, and preliminary investigations of (**6**) into γ-glutamyltranspeptidase (GTP) enzyme assay. Specific aims were to investigate a range of polymer growth conditions such as electrochemical potential windows, the number of growth cycles, ratios of novel Fc derivatives, **2** and **6** and pyrrole and 3,4-ethylenedioxythiophene (EDOT) in aqueous electrolytes. This was followed by comprehensive characterisation and assessment studies on polymers films, including their electron and charge transport properties. Finally, the redox mediation dynamics of Fc polymer films for l-glutamate were evaluated with electrochemical detection of l-glutamate and GTP. To the best of our knowledge, there is no current literature documenting the mediated electrochemistry of GTP, and the ferrocene derivatives presented here are novel and unused to date with respect to glutamate biosensing.

## 2. Experimental

### 2.1. Reagents

All solvents employed were HPLC grade, obtained from either Sigma- Aldrich or Lennox Laboratory Supplies. All ferrocene starting materials were obtained from Sigma-Aldrich. Silica gel flash chromatography was performed using 230–400 mesh silica gel. NaClO_4_, NaNO_3_, KCl, l-glutamate were acquired from Sigma and Lennox and used as received. GOx from *Aspergillus niger* (200 U mg^−1^) was purchased from Sigma. Glutamate oxidase (GlutOx) (recombinant) from cultured *E. coli.* (100 U) was obtained from Yamasa, Japan.

### 2.2. Apparatus

Cyclic voltammetry, chronoamperometry and hydrodynamic amperometry experiments were carried out using CH instruments, CHI 660 and CHI 750 potentiostats. A single-compartment electrochemical cell was used with a glassy working electrode (GCE) (area = 0.07 cm^2^), a platinum counter electrode and a reference electrode, Ag|AgCl. The latter comprised a silver wire in contact with an internal solution of KCl (3 M). Melting point analyses were carried out using a Stewart Scientific SMP1 melting point apparatus and were uncorrected. Elemental analyses were carried out in Microanalytical Laboratories of University College Dublin. Infrared spectra were obtained from a Nicolet *Impact* 410 FT-IR spectrometer using the Omnic software. Solid and liquid samples were prepared as dispersions in KBr disks and on NaCl disks, respectively. NMR spectroscopy was carried out on a Joel JNM-LA 300 FT-NMR 300 MHz and Bruker 300 and 500 MHz spectrometers using CDCl_3_ as the solvent and tetramethylsilane (TMS) as the internal standard. Chemical shifts were expressed in parts per million (δ) downfield from the internal standard.

### 2.3. Procedures

*Synthesis of 3-(1H-pyrrol-1-yl)propanoic acid* (**1**) [18]

1-(2-Cyanoethyl)pyrrole (2.40 g, 20.0 mmol) was refluxed in a solution of KOH (60 mL, 6.7 M) under argon atmosphere until production of ammonia gas ceased. The reddish-brown solution was acidified to pH 5 using HCl (8 M, 30 mL), and the product was then extracted with Et_2_O (3 × 20 mL). The ether extracts were combined and dried over anhydrous Na_2_SO_4_ and concentrated under reduced pressure to give the crude product. The product was re-crystallised twice from boiling heptane to yield **1** as white crystals (1.96 g, 71%). υ_max_/cm^−1^ 3248 (O-H), 3009 (Ar-C-H), 2889 (Alip-C-H), 1736 (C=O), 1634 (C=C) and 1339 (C-N). δ_H_ (300 MHz, CDCl_3_) 2.73 (2H, m, CH_2_ α-to C=O), 4.18 (2H, t, CH_2_ β-to C=O), 6.15 (2H, d, 2 × β-H in pyrrole), 6.64 (2H, d, 2 × α-H in pyrrole), 11.74 (OH). δ_C_ (300 MHz, CDCl_3_) 34.0 (C CH_2_ α-to C=O), 49.9 (C CH_2_ β-to C=O), 108.1 (2 × β-C in pyrrole), 122.2 (2 × α-C in pyrrole), 177.3 (COOH).

*Synthesis of 3-(1H-pyrrol-1-yl)propanamidoferrocene* (**2**)

EDC (115 mg, 0.8 mmol) was added slowly into a stirred solution of DCM (15 mL) containing 3-(1*H*-pyrrol-1-yl)propanoic acid (**1**) (70 mg, 0.5 mmol) at 0 °C. Amino ferrocene (100 mg, 0.5 mmol), diluted in a minimum amount of DCM, was degassed with argon for 5 min prior to adding into the reaction mixture. The mixture was then degassed with argon for another 5 min while stirring at 0 °C. The stirring continued in a reaction vessel wrapped in aluminium foil at room temperature under argon atmosphere for 20 h. The reaction mixture was transferred to a separating funnel and washed with HCl (3 M, 15 mL), saturated NaHCO_3_ solution and then brine. The organic phase was dried over anhydrous Na_2_SO_4_, filtered and concentrated under reduced pressure to give the crude product. The pure product was isolated by column chromatography (eluent: CHCl_3_:EtOAc, 50:50) to yield **2** as a solid orange powder (130 mg, 41%). TLC (eluent: CHCl_3_:EtOAc, 50:50) revealed a spot with R_f_ of 0.77. m.p. 130–132 °C. Found C, 64.11; H, 5.32; N, 8.21; Fe, 17.29 requires C, 63.77; H, 5.04; N, 8.75; Fe, 17.44. υ_max_/cm^−1^ 3445 (N-H), 3108 (Ar-C-H), 2915 (Alip-C-H), 1661 (C=O), 1540 (C=C) and 1323 (C-N). δ_H_ (300 MHz, CDCl_3_) 2.54 (2H, m, CH_2_ α-to C=O), 4.18 (2H, t, CH_2_ β-to C=O), 4.50 (5H, m, unsubstituted Cp), 4.71 and 5.16 (4H, d, substituted Cp), 6.17 (2H, d, 2 × β-H in pyrrole), 6.60 (2H, d, 2 × α-H in pyrrole). δ_C_ (300 MHz, CDCl_3_) 39.2 (C α-to C=O), 45.3 (C β-to C=O), 61.1 (2 × substituted Cp), 66.6 (2 × substituted Cp), 69.5 (5 × unsubstituted Fc carbons), 73.6 (Fc carbon at NH), 108.5 (2 × β-C in pyrrole), 122.4 (2 × α-C in pyrrole), 167.2 (C=O).

*Synthesis of Cyano(trimethylsilyloxy)methylferrocene* (**3**)

Trimethylsilyl cyanide (330 mg, 0.3 mmol) was added dropwise into a flask wrapped in aluminium foil containing ferrocene carboxaldehyde (550 mg, 0.3 mmol). The reaction mixture was stirred in dichloromethane (15 mL) with a catalytic amount of zinc iodide (50 mg) at room temperature under a nitrogen atmosphere overnight. Subsequently, the reaction mixture was washed with water and then saturated with NaHCO_3_ solution. The combined aqueous phase was extracted several times with DCM (3 × 15 mL). The organic phase was combined, dried over anhydrous Na_2_SO_4_ and filtered. The filtrate was concentrated under reduced pressure to afford a reddish-brown solid (750 mg, 97%). TLC (eluent: DCM) revealed a spot with R_f_ of 0.85. υ_max_/cm^−1^ 3113 (Ar-C-H), 2922 (Alip-C-H), 2278 (C≡N), 1687 (C=C) and 1083 (C-O). δ_H_ (300 MHz, CDCl_3_) 1.25 (9H, s, Me_3_-Si), 4.28 (5H, m, Cp), 4.61 and 4.80 (4H, d, substituted Cp), 5.32 (1H, s, CH(OTMS)CN). δ_C_ (300 MHz, CDCl_3_) 0.98 (CH_3_), 62.3 (CH(OH)), 67.3 (2 × substituted Cp), 68.8 (2 × substituted Cp), 69.1 (5 × unsubstituted Cp), 84.8 (Cp substituted by CH(SiMe_3_CN)), 118.5 (C≡N).

*Synthesis of 2-amino-1-hydroxyethylferrocene* (**4**)

Solid LiAlH_4_ (116 mg, 3 mmol) was added slowly to a solution of (**3**) (300 mg, 1.0 mmol) in anhydrous Et_2_O (10 mL). The reaction mixture was stirred at room temperature under a nitrogen atmosphere overnight. Excess LiAlH_4_ was quenched with the careful addition of an ethanol: Et_2_O mixture (50:50) (5 mL). The Et_2_O layer was washed successively with HCl (2 M, 2 × 5 mL), NaOH (5 mL, 2 M) and water. The product was dried over anhydrous MgSO_4_, the solvent was removed under reduced pressure and the product was then isolated by column chromatography (eluent, DCM:MeOH, 90:10) to yield (**4**) as a yellow solid (55 mg, 23%).

TLC (eluent: DCM:MeOH, 90:10) revealed a spot with R_f_ of 0.25. Found C, 59.86; H, 5.46; N, 5.21; Fe, 22.49 requires C, 59.29; H, 5.39; N, 5.76; Fe, 22.97. υ_max_/cm^−1^ 3537 (O-H), 3362 (N-H), 3132 (Ar-C-H), 2932 (Alip-C-H) and 1663 (C=C). δ_H_ (300 MHz, CDCl_3_) 2.76 and 3.00 (2H, d, CH_2_-NH_2_), 4.12 (5H, m, Cp), 4.20 and 4.35 (4H, d, substituted Cp), 4.53 (1H, t, CH-OH). δ_C_ (300 MHz, CDCl_3_) 48.4 (CH_2_-NH_2_), 66.9 (2 × substituted Cp), 67.9 (2 × substituted Cp), 69.6 (5 × unsubstituted Cp), 72.2 (CH-OH) 91.0 (Cp substituted by CH(OH)).

*Synthesis of 3-(2-bromoethyl)thiophene* (**5**)

A mixture of 2-(thiophen-3-yl)ethanol (128 mg, 1.0 mmol) and triphenylphosphine dibromide (Ph_3_PBr_2_) (260 mg, 1.0 mmol) was refluxed in anhydrous CH_3_CN (20 mL) under nitrogen atmosphere overnight. Upon cooling, the product mixture was filtered and concentrated under reduced pressure. Afterwards, Et_2_O (100 mL) was added, the product mixture was filtered again and washed with distilled water to its pH at neutrality. The organic layer was separated and dried over anhydrous Na_2_SO_4_ and concentrated under reduced pressure. The residue was purified by column chromatography (silica, heptane) to yield a yellow oil (125 mg, 65%).

TLC (eluent: hexane: DCM, 80:20) revealed a spot with R_f_ of 0.68. υ_max_/cm^−1^ 3136 (Ar-C-H), 2879 (Alip-C-H), 1628 (C=C), 1155 (C-S) and 530–603 (C-Br). δ_H_ (300 MHz, CDCl_3_) 3.02 (2H, t, H_b_), 3.56 (2H, t, H_a_), 6.69 and 6.72 (2H, m, H_c_ and H_e_) and 7.03 (1H, d, H_d_). δ_C_ (300 MHz, CDCl_3_) 32.3 and 32.4 (C_a_ and C_b_), 121.3 (C_e_), 126.1 (C_d_), 128.1 (C_c_) and 138.9 (C-substituted thiophene).

*Synthesis of 1-hydroxy-2-[2-(thiophen-3-yl)-ethylamino]ethylferrocene* (**6**)

A mixture of (2-amino-1-hydroxyethyl)ferrocene (**5**) (120 mg, 0.5 mmol), 3-(2-bromoethyl)thiophene (96 mg, 0.5 mmol) and anhydrous K_2_CO_3_ (137 mg, 1.0 mmol) was refluxed in anhydrous DMF (5 mL) under nitrogen atmosphere for 48 h. The product mixture was then cooled, and the solvent was removed under reduced pressure. The crude product was diluted with EtOAc (10 mL) and washed with water and brine. The organic layer was dried over anhydrous Na_2_SO_4_ and concentrated under reduced pressure. The product was isolated by column chromatography (eluent, DCM:MeOH, 90:10) to yield **6** as an orange oil (224 mg, 63%). TLC (eluent: DCM:MeOH, 90:10) revealed a spot with R_f_ of 0.56. Found C, 61.57; H, 5.07; N, 4.06; Fe, 15.48; requires C, 61.20; H, 5.42; N, 3.96; Fe, 15.81. υ_max_/cm^−1^ 3585 (O-H), 3361 (N-H), 3127 (Ar-C-H), 2962 (Alip-C-H), 1633 (C=C) and 1133 (C-S). δ_H_ (300 MHz, CDCl_3_) 2.82 (2H, t, CH_2_ α-to thiophene ring), 2.88 (2H, t, CH_2_ β-to thiophene ring), 2.90 and 3.15 (2H, d, CH_2_ β-to Fc ring), 4.15 (5H, m, unsubstituted Cp), 4.21 and 4.29 (4H, d, substituted Cp), 4.32 (1H, t, CH-OH), 6.74 and6.76 (2H, m, thiophene H-2 and H-4) and 7.27 (1H, d, thipohene H-5). δ_C_ (300 MHz, CDCl_3_) 30.9 (C α-to thiophene ring), 50.1 (C β-to thiophene ring), 55.0 (C β-to Fc ring), 67.3 (2 × Cp), 67.7 (2 × substituted Cp), 70.1 (5 × unsubstituted Cp), 73.2 (CH-OH), 90.8 (Fc substituted by CH(OH)), 121.3 (C-2), 126.2 (C-4), 128.4 (C-5), 141.2 (C-substituted thiophene).

Preparation of Glutamate oxidase electrodes

GlutOx (100 U) and BSA (10 mg) were dissolved in 150 µL of PBS pH 7.2 (10 mM) containing KCl (10 mM). A total of 5 µL of the solution was then mixed with 1.65 µL of Nafion (0.5%) and 3.3 µL of glutaraldehyde (2.5%). The mixture was manually deposited by pipette onto the polished electrode surface and left at ambient temperature for about 2 h to dry/crosslink. The enzyme electrode (GlutOx ~3 U) was then stored in PBS at 4 ºC overnight to equilibrate.

Solution-mediated electrochemical detection of l-glutamate

A solution of Fc derivative (1 mM) in PBS pH 7.2 (10 mM) containing KCl (10 mM) underwent cyclic voltammetry at 10 mV s^−1^ at a GlutOx modified GCE vs. Ag|AgCl. Increases in electrocatalytic current responses to incremental l-glutamate additions (5 mM) were measured relative to the background current and referred to as catalytic waves. In the case of hydrodynamic amperometry, a solution of Fc derivative (1 mM) in PBS pH 7.2 (10 mM) containing KCl (10 mM) was analysed at a GlutOx modified GCE at a constant applied potential (E_app_) (depending on the Fc derivative used) vs. Ag|AgCl. After having allowed a period of 10 min for the background current to be stabilised, increases in amperometric current responses to incremental l-glutamate additions (0.5 mM) in the solution at every 5 min were measured relative to the background current and referred to as steady-state currents.

Electropolymerisation of Fc derivatives and Pyrrole/EDOT by Potential Sweeping

LiClO_4_ aqueous solution (10 mM, 3 mL) containing Fc derivative (1–5 mM) and pyrrole/EDOT (0.1–5 mM) were cycled at 100 mV s^−1^ for 2 cycles vs. Ag|AgCl on the GCE surface between −0.20 V and +1.20 V for 3-(1*H*-pyrrol-1-yl)propanamidoferrocene (**2**) and 1-hydroxy-2-[2-(thiophen-3-yl)-ethylamino]ethylferrocene (**6**) and pyrrole using CV. A potential range between 0 V and +1.20 V was employed for **2** and **6** and EDOT electropolymerisations. Following polymer growth, the modified electrodes were rinsed thoroughly with fresh aqueous LiClO_4_ solution (10 mM) to remove any unpolymerised material. All the films were then stabilised in LiClO_4_ solution (10 mM) by cycling between 0 V and 0.60 V. A potential range between −0.10 V and 0.50 V was used for those of **2** and pyrrole for 25 repeated cycles at 25 mV s^−1^. A multiple scan rate study was then performed for the films over the scan rate range 1–1000 mV s^−1^ in a fresh solution of aqueous LiClO_4_ (10 mM) over the same potential limits.

Mediated electrochemical detection of l-glutamate

The resulting polymer films were cycled in PBS pH 7.2 (10 mM) containing KCl (10 mM) at 0.5–10 mV s^−1^ at a GlutOx modified electrode vs. Ag|AgCl. Electrocatalytic responses to incremental l-glutamate additions in solution (up to 5 mM) were monitored. In the case of chronoamperometric studies, a single step of a pulse width of 60 s and sample interval of 0.063 s was applied to the resultant polymer films in PBS pH 7.2 (10 mM) containing KCl (10 mM) vs. Ag|AgCl at a GlutOx modified GCE between redox potential range (depending on the Fc derivative used). Electrocatalytic responses to incremental l-glutamate additions were monitored.

Unmediated electrochemical detection of γ-glutamyltranspeptidase using cyclic voltammetry and chronoamperometry

A solution of γ-l-glutamyl-l-glutamate (10 mM) and glycyl-glycine (200 mM) in PBS pH 7.2 (10 mM) containing KCl (10 mM) was analysed by cycling at 10 mV s^−1^ at a GlutOx modified platinum working electrode within a potential range of 0–1 V vs. Ag|AgCl. Increases in electrocatalytic current responses to incremental GTP additions (100 U/L) in solution were measured relative to the background current. A single step of a pulse width of 60 s and sample interval of 0.063 s was applied to a solution of γ-l-glutamyl-l-glutamate (10 mM) and glycyl-glycine (200 mM) in PBS pH 7.2 (10 mM) containing KCl (10 mM) at a GlutOx-modified platinum working electrode between a potential range of 0–0.70 V vs. Ag|AgCl. Increases in electrocatalytic current responses to incremental GTP additions (100 U/L) in solution were measured relative to the background current.

Mediated electrochemical detection of GTP using cyclic voltammetry

A solution containing Fc derivative (1 mM), γ-l-glutamyl-l-glutamate (10 mM) and glycyl-glycine (200 mM) in PBS pH 7.2 (10 mM) containing KCl (10 mM) was cycled at 10 mV s^−1^ at a GlutOx modified GCE. Increases in electrocatalytic current responses to cumulative GTP additions (100 and 500 U/L) in solution were measured relative to the background current.

## 3. Results and Discussion

**Scheme 1:**
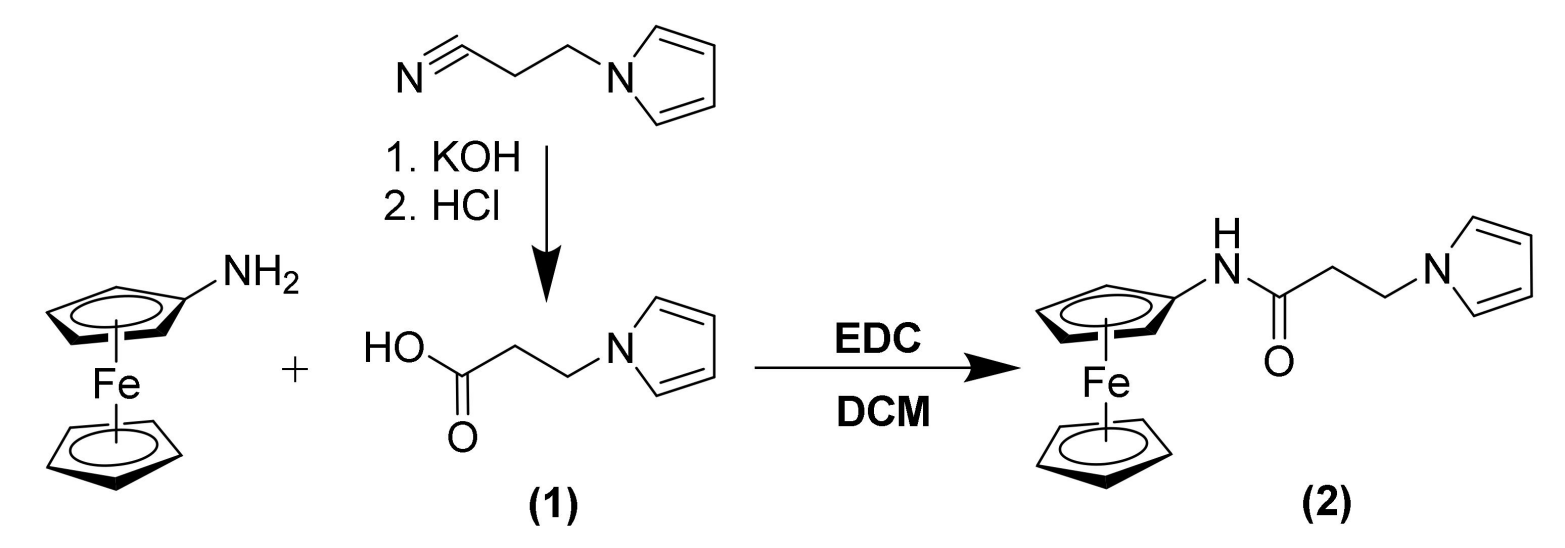
Synthesis of 3-(1*H*-pyrrol-1-yl)propanamidoferrocene (**2**).

**Scheme 2:**
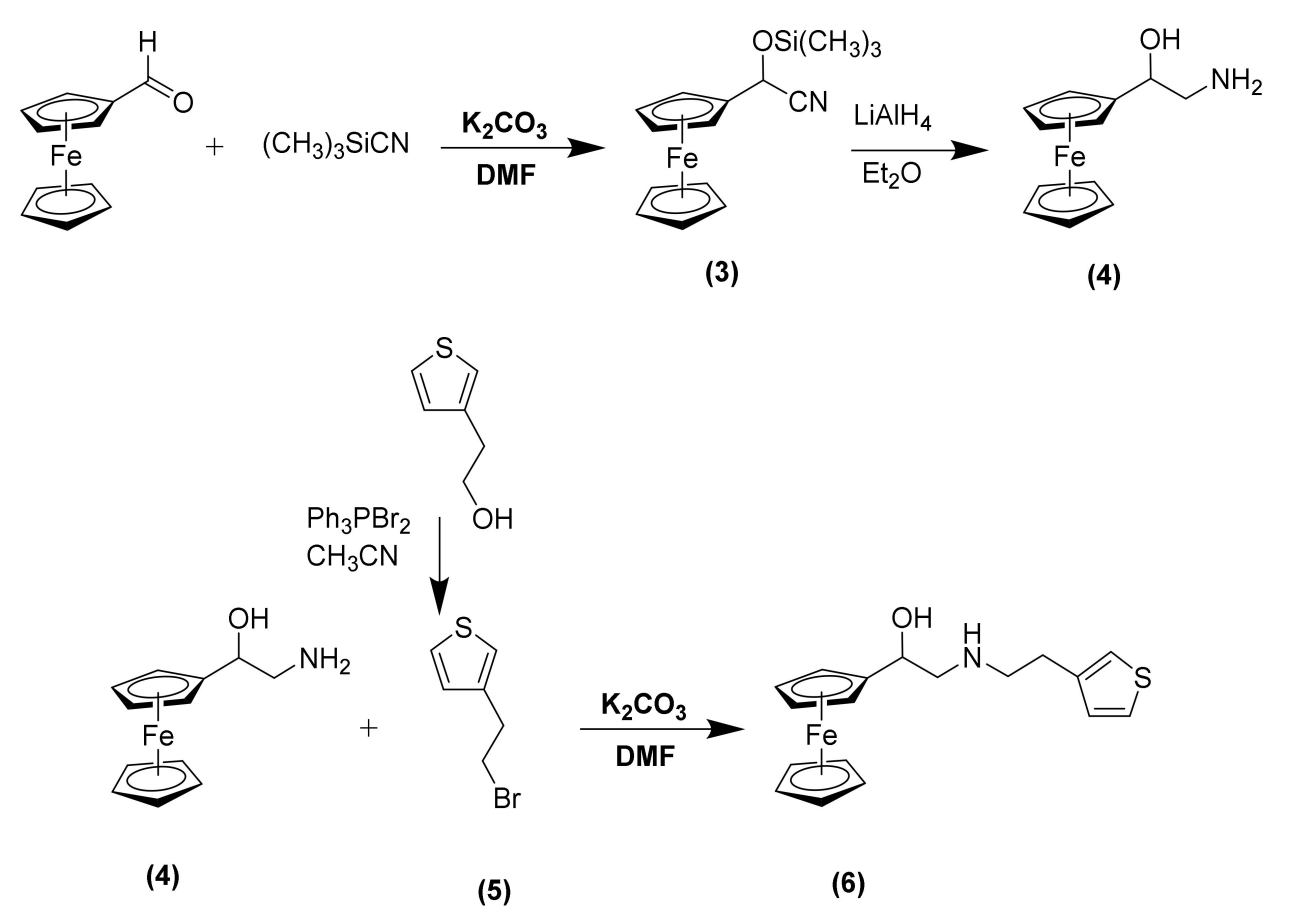
Synthesis of 1-hydroxy-2-[2-(thiophen-3-yl)-ethylamino]ethylferrocene (**6**).

Two new ferrocene (Fc) derivatives linked by methylene groups to pyrrole and thiophene functionalities, respectively, have been prepared according to the synthetic routes described in Scheme 1 and Scheme 2, respectively.

The ferrocene compounds used as starting materials, namely, aminoferrocene and ferrocene carboxyaldehyde, were commercially available. The design of the Fc derivatives in this study was influenced by the published literature, as summarised below. Fc compounds are an important mediator class to ensure extremely low levels of enzymes; their fragments and products from redox processes can be detected accurately in commercial biosensors, as mentioned earlier. Polypyrrole polymer networks, where polymerisation occurs through the α-carbon to the N, are known to immobilise enzymes such as glutamate oxidase [10]. The attachment of the Fc to the 5-membered heterocyclic analogue by a short chain is also important. A comprehensive report by Forrow et al. [19] described how the electronic effects tend to diminish when electron-donating or -withdrawing substituents are too far (n > 3 or 4) from the Cp ring. The hydrophilicity of the Fc derivatives also depends on the nature of the substituents on the Cp ring. In addition, Tustin et al. [20] highlighted a shift of the E_1/2_ of the Fc derivatives substituted with electron-donating substituents, such as methyl- or amino- groups, in the cathodic direction. Fc derivative **2** was designed with the amine moiety directly attached to the ring to achieve lower redox potential and higher hydrophilicity.

Thiophenes are 5-membered ring heterocyclic structures that are also capable of polymerisation at the α-carbon. Polythiophenes substituted at the β-carbon offer an excellent alternative to polypyrroles for use in sensing materials due to their low cost, ease of processing and high thermal stability [21]. Polythiophene boronic acids on chitosan have been used to immobilise enzymes for glucose sensing [22]. However, an overview of techniques in enzyme immobilisation carried out in 2017 indicates that their use is rare in biosensor polymer networks currently [23]. Polythiophenes are more utilised as biocatalysts for other applications [24].

3-(1*H*-pyrrol-1-yl)propanoic acid (**1**) was produced by refluxing 1-(2-cyanoethyl)pyrrole in KOH following the known literature experimental procedure for nitrile hydrolysis, with subsequent acidification of the salt to give compound (**1**) as a white crystalline solid in high yield, following purification by recrystallisation. Amino ferrocene was then coupled to the pyrrole carboxylic acid compound (**1**), which was first activated with 1-Ethyl-3-(3-dimethylaminopropyl)carbodiimide (EDC), to produce the required (**2**) as a solid, following purification by column chromatography.

The Fc thiophene analogue (**6**) was synthesised in a multi-step synthesis, starting with ferrocene carboxaldehyde. The aldehyde was treated with trimethylsilyl cyanide in dichloromethane solvent with ZnI_2_ as the catalyst to effect the addition of the silyl cyanide across the carbonyl double bond to obtain the cyano TMS addition adduct (**3**) as a solid in nearly quantitative yield. Reduction of the nitrile was effected with an excess of LiAlH_4_ in diethyl ether as a solvent, which also resulted in cleavage of the Si-O bond to yield the amino hydroxyethyl ferrocene derivative (**4**) as a yellow solid following purification by column chromatography. The alkylating agent 3-(2-bromoethyl)thiophene (**5**) was synthesised from the commercially available 3-(2-hydroxyethyl)thiophene by substitution involving triphenylphosphine dibromide as a brominating agent in acetonitrile at reflux following the procedure of [25]. (**6**) was obtained by alkylation of 2-amino-1-hydroxyethyl) ferrocene (**4**) with 3-(2-bromoethyl)thiophene (**5**) and a twofold excess of anhydrous K_2_CO_3_ in anhydrous DMF at reflux to yield the required compound as an oil following purification by column chromatography.

The synthesised Fc derivatives allowed an investigation into their redox potentials and hydrophilicity for potential applications as redox mediators in glutamate sensing with extension to proof-of-principle GTP enzyme assay. Both thiophene- and pyrrole-based conducting polymers have found excellent use in biosensing applications, and thus Fc derivatives incorporating both heterocycles have been the key focus in this work.

### Solution Phase Mediated Electrochemical Detection of l-Glutamate

Initial solution studies of glutamate quantitation at each Fc derivative was examined with mediated electrochemical detection of l-glutamate according to:

Glutamate + 2Fc^+^ → α-ketoglutarate + NH_3_ +2Fc 2Fc → 2Fc^+^ + 2e^−^

Mediated detection of l-glutamate using (3-(1H-pyrrol-1-yl)propan amido)ferrocene (2) and 1-hydroxy-2-[2-(thiophen-3-yl)-ethylamino]ethylferrocene (6)

**Figure 1:**
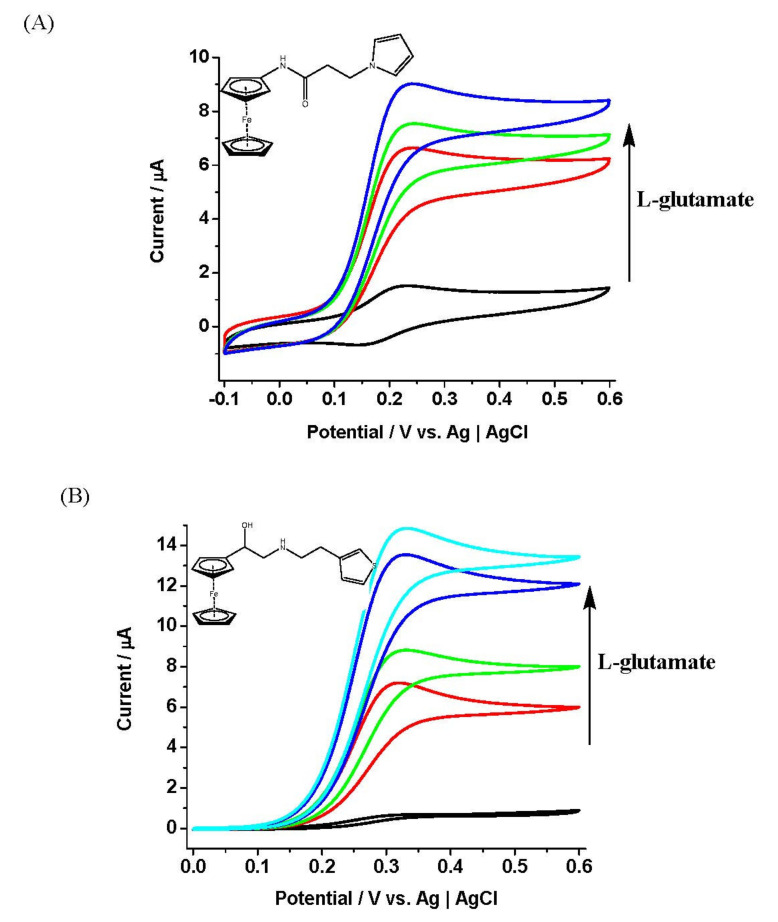
(**A**) Cyclic voltammetry responses of (3-(1*H*-pyrrol-1-yl)propanamido) ferrocene (**2**) (1 mM) towards successive additions of l-glutamate (0–40 mM) at a GlutOx electrode. Solution: PBS pH 7.2 (10 mM)-KCl (10 mM) (black curve). Potential sweep, 0–+0.6 V vs. Ag|AgCl, scan rate 10 mV s^−1^. (**B**) Cyclic voltammetry responses of (1-hydroxy-2-(2-(thiophen-3-yl)ethyl amino)ethyl)ferrocene (**6**) (1 mM) towards successive additions of l-glutamate (0–20 mM) at a GlutOx electrode. Solution: PBS pH 7.2 (10 mM)-KCl (10 mM) (black curve). Potential sweep, 0–+0.6 V vs. Ag|AgCl, scan rate 10 mV s^−1^.

Figure 1A shows the cyclic voltammetry response increases of (3-(1*H*-pyrrol-1-yl) propanamido)ferrocene (**2**) (1 mM) in PBS solution at a GlutOx electrode with successive l-glutamate additions (5 mM) up to 15 mM. Experiments with this **2**-GlutOx electrode were also conducted at very low scan rates, giving rise to steady-state catalytic currents of the order of 0.76 µA/mM (SI 1(A)). In CA measurements (SI 2(A)), the catalytic current response of the **2**-GlutOx electrode increased with l-glutamate concentration beyond 15 mM, and the glutamate sensitivity measured by CA was 0.52 µA/mM. Hydrodynamic amperometry (HA) of the **2**-GlutOx electrode, at an applied potential of +0.25 V, displayed anodic current responses, which increased in a near-linear manner with l-glutamate concentration, <5 mM (SI 3(A)). Glutamate sensitivity of the **2**-GlutOx electrode as measured by HA was of the order of 0.86 µA/mM. Figure 1B shows the cyclic voltammetry response of (**6**) (1 mM) towards consecutive increments of l-glutamate (5 mM) up to 20 mM at a GlutOx electrode in PBS solution. Close to a steady-state condition is achieved in this case at a relatively fast scan rate of 10 mV s^−1^. The voltammetric study of the **6**-GlutOx electrode was extended at slower scan rates, resulting in steady-state catalytic currents of the order of 0.83 µA/mM (SI 1(B)). CA data (SI 2) indicates that catalytic currents for the **6**-GlutOx and 2-GlutOx electrodes increased linearly with l-glutamate concentrations < 11 mM. The I/C sensitivity of the GlutOx electrode to glutamate measured by CA was 0.92 µA/mM. Further amperometric experiments at a fixed potential of +0.30 V revealed that current responses increased linearly with l-glutamate at <3.5 mM and plateaued when >9 mM. Glutamate sensitivity of the **6**-GlutOx electrode as measured by HA was of the order of 1.28 µA/mM (SI 3(B)).

GlutOx electrode kinetic parameters were estimated from hydrodynamic amperometry data, resulting in I_max_ 21.89 μA (**2**), 11.80 μA (**6**), K_m_ 5.01 mM (**2**) and 3.69 mM (**6**) with E_1/2_ values 0.18 (**2**) and 0.26 V (**6**) and sensitivity 0.86 µA mM^−1^ (**2**) and 1.28 µA mM^−1^ (**6**). These Fc derivatives displayed very good solubility in aqueous media and good analytical performance towards l-glutamate. They also possess low E_1/2_ values, being advantageous, as natural-occurring interferences in real biological samples can be avoided.

Fc derivatives **2** and **6** were next copolymerised with pyrrole and EDOT monomer, respectively, to produce electroactive conducting films. An optimisation study on polymer growth conditions including growth cycles, potential range, monomer and Fc derivatives ratios was conducted to assess the redox behaviour exhibited by the resulting copolymer films. An assessment of the mediation capabilities of these films towards l-glutamate was then conducted in the presence of immobilised oxidases. Homopolymerisation of Fc derivatives **2** and **6** was attempted with varied anodic limits, without success. These observations were in agreement with literature reports about ineffective homopolymerisation of Fc derivatives bearing bulky groups close to the heteroaromatic rings. The film preparation procedure here comprised of varied experimental conditions such as ratios of Fc derivatives and pyrrole/EDOT of 1 mM: (1–5 mM) over different growth cycles (1–5 cycles). The aim was to establish the optimised conditions for film growth and their effect on the redox behaviour of the resulting films towards l-glutamate. Potential limits for polymer growth and stabilisation were also optimised. Following polymer growth, the films were washed with fresh aqueous media to remove any unbound monomer and then stabilised by repeated cycles using CV in a fresh solution of aqueous media containing LiClO_4_. A range of scan rates and potential windows over varied cycles were employed to determine the conditions necessary for stabilisation. Afterwards, multiple scan CV experiments were carried out on the films to assess the dynamics of heterogeneous ET across the electrode/film layer interface.

Polymerisation of 3-(1*H*-pyrrol-1-yl)propanamidoferrocene (2) and pyrrole using cyclic voltammetry

**Figure 2:**
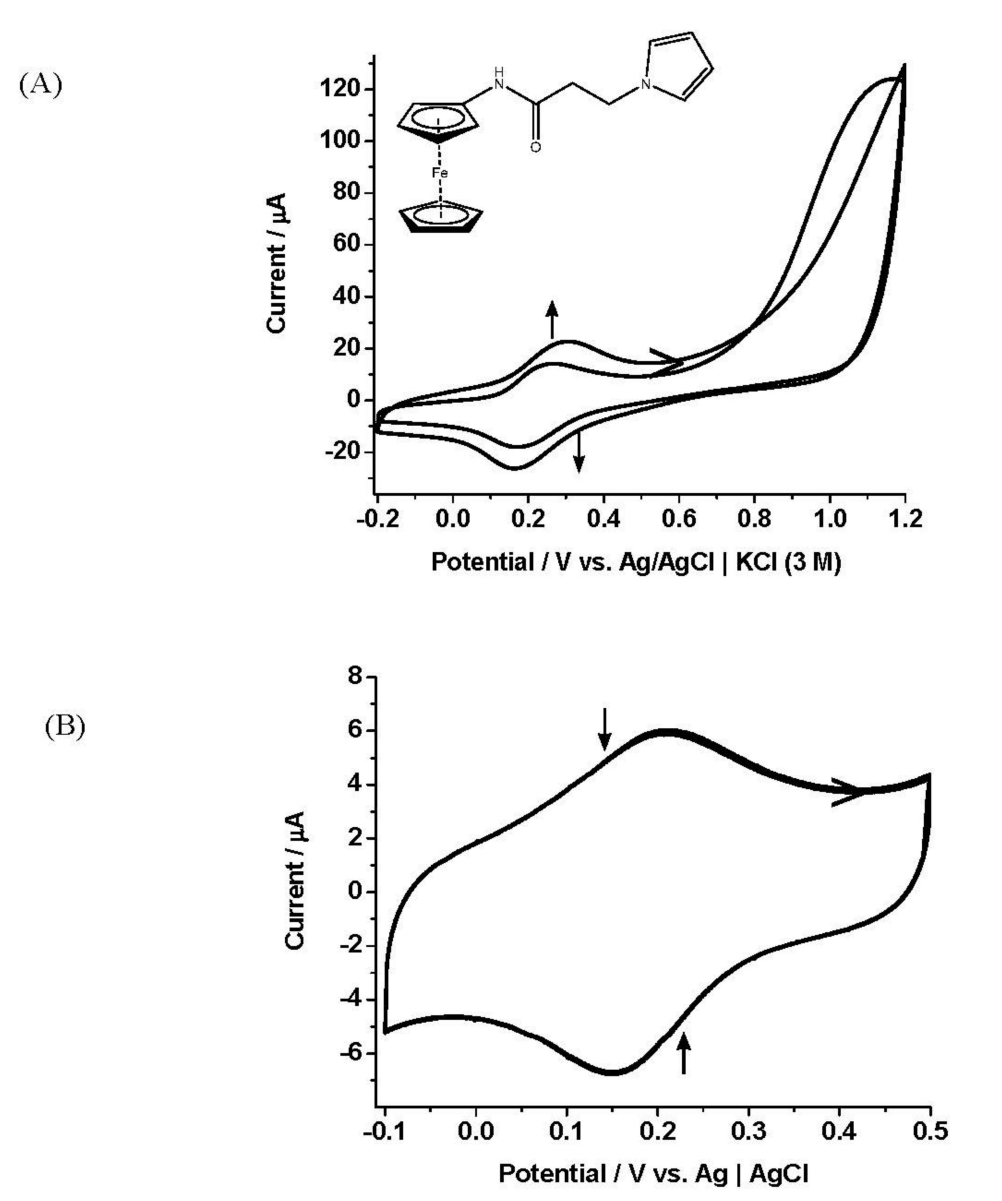

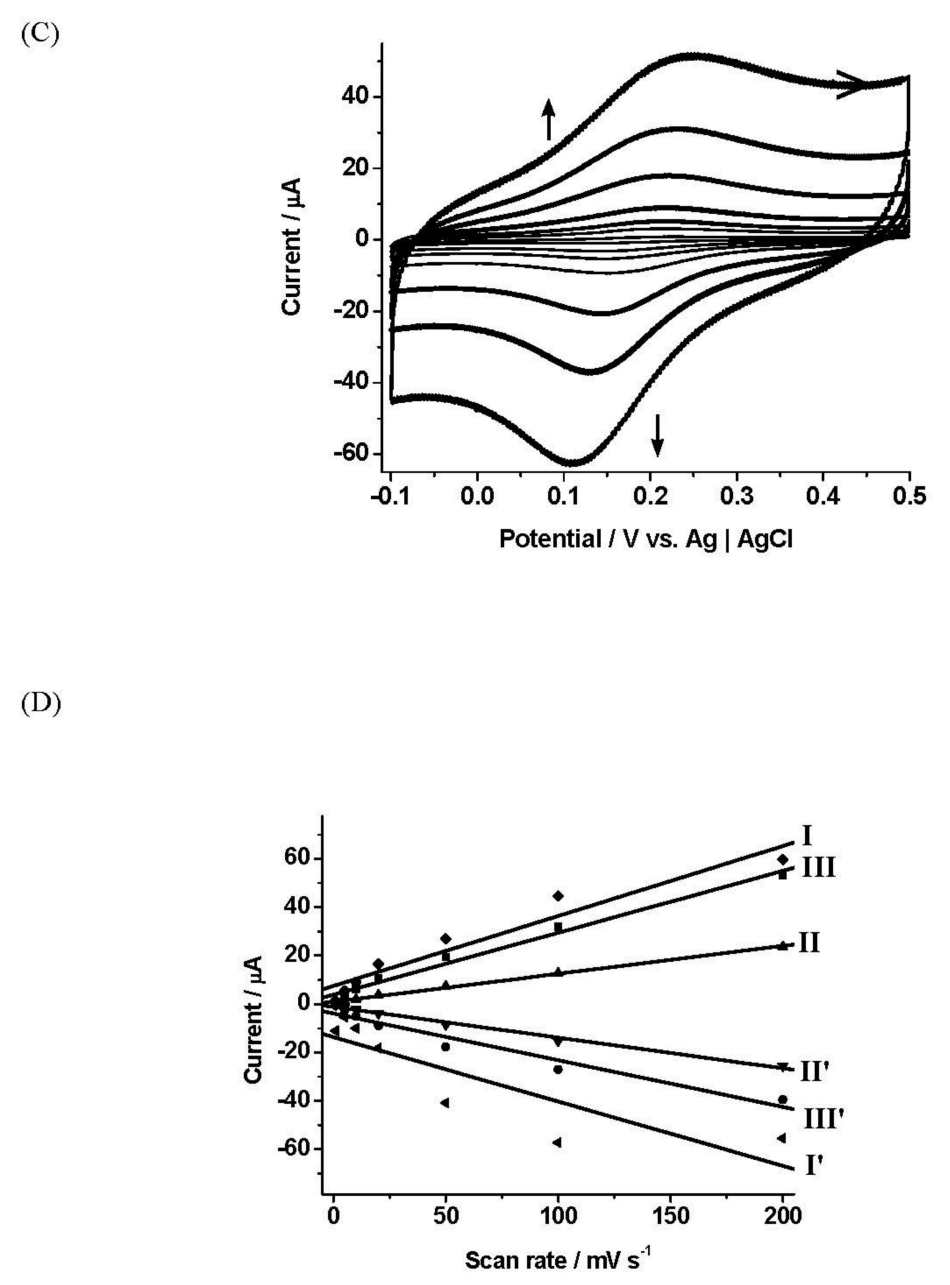
(**A**) Polymerisation of 3-(1*H*-pyrrol-1-yl)propanamidoferrocene (**2**) and pyrrole in ratio of 1:5 (1 mM:5 mM) at a GCE in aqueous LiClO_4_ (10 mM). Potential sweep, 0–+1.20 V vs. Ag|AgCl, scan rate 100 mV s^−1^. (**B**) Stability of the films of 2-pyrrole in ratio of 1:5 (1 mM:5 mM) at a GCE in the same supporting electrolyte. Potential sweep, −0.10–+0.50 V vs. Ag|AgCl, scan rate 25 mV s^−1^ for 25 cycles. (**C**) Variable scan rate study of the films of 2-pyrrole in ratio of 1:5 (1 mM:5 mM) at a GCE. Potential sweep, −0.10–+0.50 V vs. Ag|AgCl, scan rate of 1–200 mV s^−1^ for 3 cycles. (**D**) *i*_p_ (μA) vs. scan rate (mV s^−1^) for the films of 2-pyrrole. I and I’ = *i*_pa_ and *i*_pc_ of pulse width of 600 s; II and II’ = *i*_pa_ and *i*_pc_ of pulse width of 800 s; III and III’ = *i*_pa_ and *i*_pc_ of pulse width of 1000 s.

Here we discuss the film formation of Fc derivative **2** and pyrrole in aqueous LiClO_4_ solution (10 mM) by anodic oxidation yielded electroactive conducting films using CV. No occurrence of polymer growth using lower monomer ratios was observed. Polymer growth of **2** and pyrrole in different ratios (1 mM: (1 mM–5 mM)) over two cycles is presented. A detailed study on assessing the effect of growth cycles and potential windows from −0.20 V to +1.20–1.60 V on the film quality was conducted. No polymer growth was found for one growth cycle. Higher growth cycles produced films of significantly higher Γ and *i_p_* loss values. Optimised growth conditions were found to be −0.20 V to +1.30 V over two growth cycles. Figure 2A presents polymer growth of 2 and pyrrole in a ratio of 1:5 (1 mM:5 mM) over two cycles in aqueous LiClO_4_ solution (10 mM). The Fe ^2+/3+^ redox process was evident at E_1/2_ = 179 mV vs. Ag|AgCl. The anodic current peaked at +0.10–0.30 V and +0.70–1.20 V due to redox oxidation of Fc to Fc^+^ in **2** and pyrrole, respectively. The current at +0.20 V in the second polymer growth cycle was higher as the conducting polymer on the electrode surface was already formed during the initial cycle. Figure 2B presents the films of **2**-pyrrole in ratio 1:5 (1 mM:5 mM), being stabilised by repetitive cycling. The E_1/2_ value for the immobilised species Fe^2+/3+^ was 205 mV vs. Ag|AgCl, resulting in a 26 mV positive shift relative to the solution electrochemistry for **2**. This E_1/2_ shift is probably due to the electrostatic influence of the positively charged PPy. Figure 2C illustrates the films made of **2** and pyrrole in the ratio of 1:5 (1 mM:5 mM) in a variable scan rate study. The current increased linearly with scan rates up to 200 mV s^−1^. At 100–200 mV s^−1^, the anodic and cathodic peaks diverged in positive and negative directions, respectively, causing increases in ΔE_p_ values, indicating semi-infinite diffusion behaviour. Figure 2D shows plots of *i*_p_ (μA) vs. scan rate (mV s^−1^) of the films of **2** and pyrrole in different ratios, prepared using CV. The films exhibited favourable linear dependence of the current on scan rates up to 200 mV s^−1^. The slopes of the films formed in ratio 1:1 (1 mM:1 mM) were the steepest, suggesting fast anion insertion and ejection rates within these films.

The films here displayed lower overall *i_p_* loss values 4.53–7.82%, and E_1/2_ values were consistent with scan rates, an indication of excellent stability. Moreover, films showed overall higher FWHM_ox_ (full width at half peak maximum) values than FWHM_red_, indicating a more facile cathodic process within the films (Table 1).

SI 4(A) displays a Laviron plot of ΔE_p_ vs. log υ for the films of **2**-pyrrole in a ratio of 1:5 (1 mM:5 mM) produced using CV. Irreversible electrochemistry was observed when ΔE_p_ > 200 mV at the scan rate of 10 mV s^−1^. The values of D_CT_^½^C, α and *k_s_* are summarised in Table 2.

Polymerisation of 1-hydroxy-2-[2-(thiophen-3-yl)-ethylamino]ethylferrocene (6) and EDOT using cyclic voltammetry

**Figure 3:**
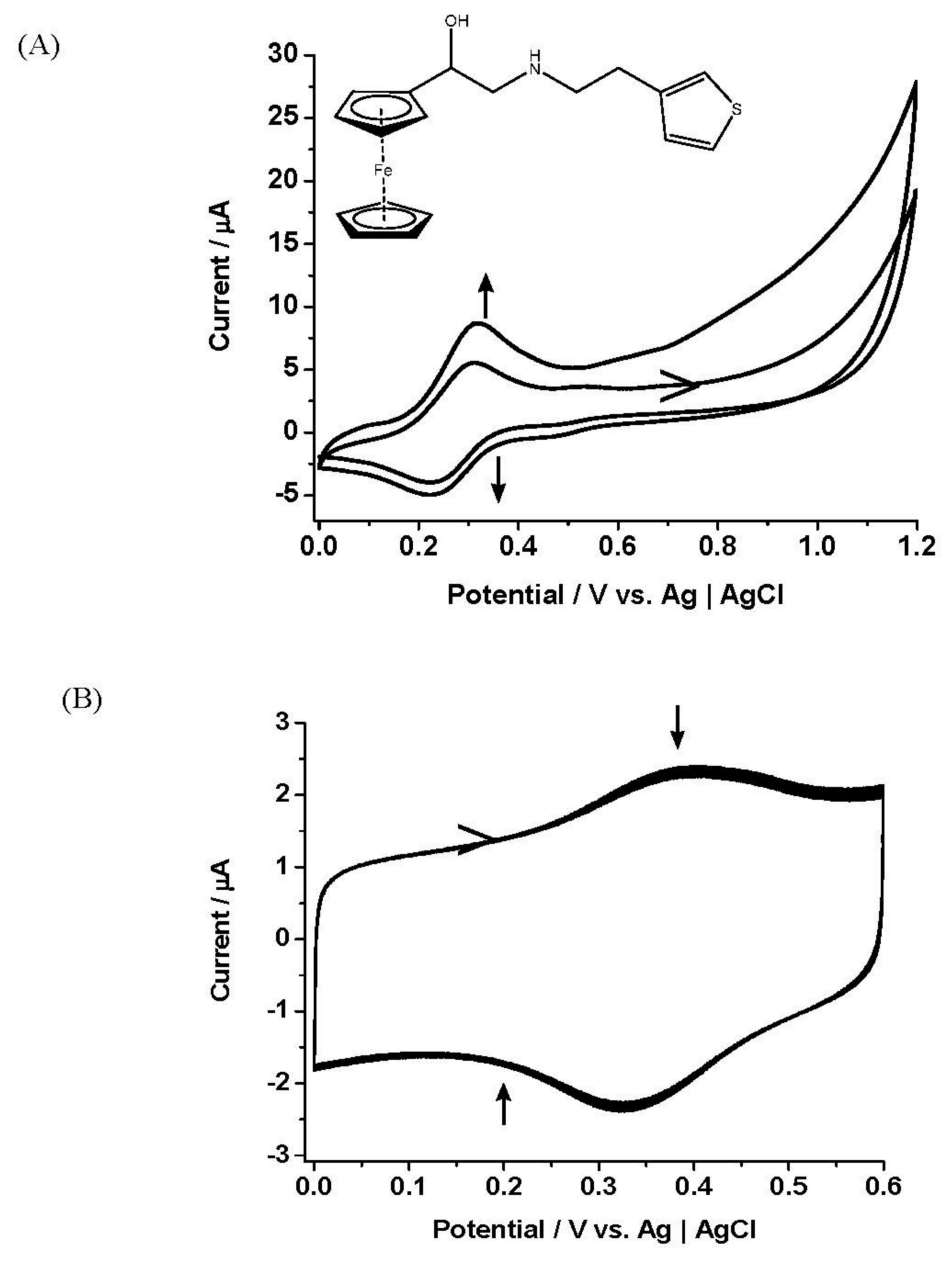

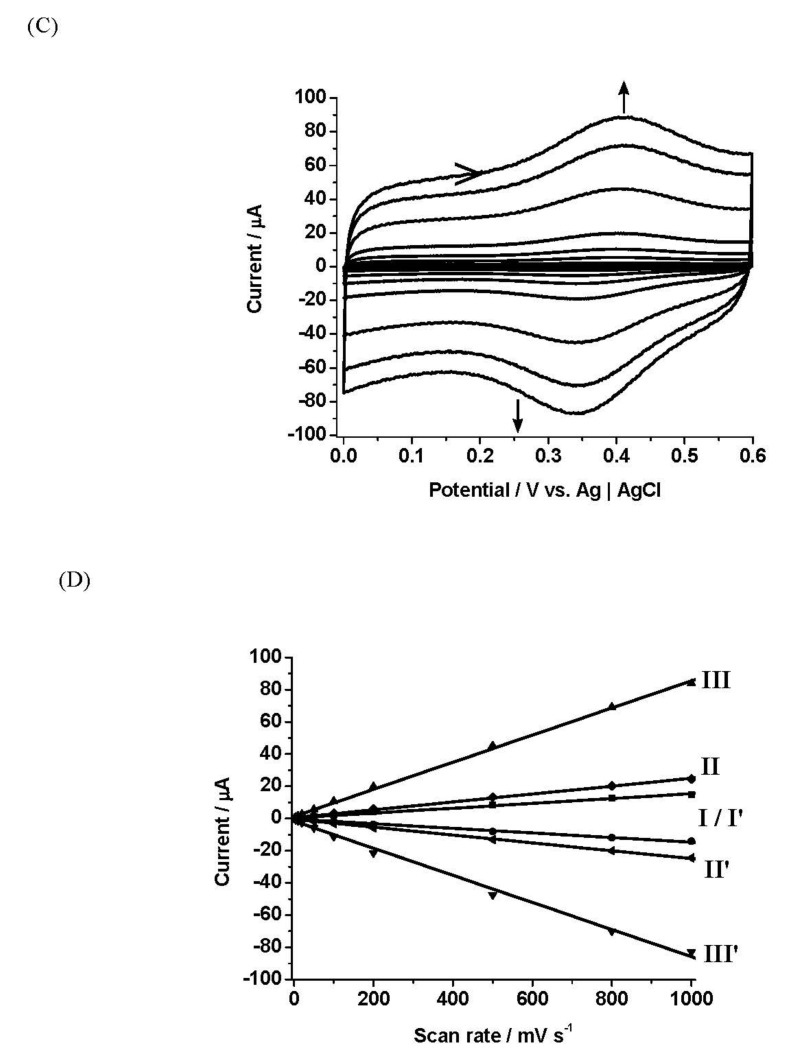
(**A**) Polymerisation of 3-(1*H*-pyrrol-1-yl)propanamidoferrocene (**6**) and EDOT in ratio of 1:5 (1 mM:5 mM) at a GCE in aqueous LiClO_4_ (10 mM). Potential sweep, 0–+1.20 V vs. Ag|AgCl, scan rate 100 mV s^−1^. (**B**) Stability of the films of 16-EDOT in ratio of 1:5 (1 mM:5 mM) at a GCE in supporting electrolyte. Potential sweep, 0–+0.60 V vs. Ag|AgCl, scan rate 25 mV s^−1^ for 25 cycles. (**C**) Multiple scan rate study of the films of 6-EDOT in ratio of 1:5 (1 mM:5 mM) at a GCE. Potential sweep, 0–+0.60 V vs. Ag|AgCl, scan rate of 1–1000 mV s^−1^ for 3 cycles; (**D**) *i*_p_ (μA) vs. scan rate (mV s^−1^) for the films of 6-EDOT. I and I’ = *i*_pa_ and *i*_pc_ in ratio of Fc:EDOT 1:1 (1 mM:1 mM); II and II’ = *i*_pa_ and *i*_pc_ in ratio of Fc:pyrrole 1:2.5 (1 mM:2.5 mM); III and III’ = *i*_pa_ and *i*_pc_ in ratio of Fc:pyrrole 1:5 (1 mM:5 mM).

Films formed with different ratios of **6** and EDOT (1 mM:(1–5 mM)) in aqueous LiClO_4_ solution (10 mM) using CV were then examined. Polymer growth using lower monomer ratios was attempted but not successful. Polymer growth of **6** and EDOT in different ratios (1 mM:(1 mM–5 mM)) over two growth cycles using CV is present here. No polymerisation was observed over one growth cycle, while two growth cycles delivered very stable films with low *i_p_* loss and Γ values. Potential windows from 0 V to +1.20–1.60 V were employed to establish optimised polymer growth conditions; 0 V to +1.20 V produced the most stable films. Figure 3A presents polymer growth of **6** and EDOT in a ratio of 1:5 (1 mM:5 mM) in aqueous LiClO_4_ solution (10 mM) with its evident redox process of Fe^2+/3+^ at E_1/2_ = 275 mV vs. Ag|AgCl. The anodic current was observed to peak at +0.30 V and 1.00 V due to electrochemical oxidation of Fc to Fc^+^ and thiophene moiety and EDOT monomer, respectively. The anodic current at +0.30 V during the second polymer growth cycle was observed to be higher as conducting polymer had already formed in the initial cycle.

Figure 3B displays the films formed in ratio 1:5 (1 mM:5 mM), being stabilised, subsequent to polymer growth, by repetitive cycling. The E_1/2_ value for the immobilised species Fe^2+/3+^ was 0.367 V vs. Ag|AgCl, resulting in a 92 mV positive shift relative to the solution electrochemistry for **6**. The currents appreciated and depreciated above zero current. This was associated with the electrode surface being rendered conductive by PEDOT formation. Figure 3C presents a multiple scan rate study of the films of **6**-EDOT in the ratio of 1:5 (1 mM:5 mM). Well-defined peaks were observed, which increased linearly with scan rates up to 1000 mV s^−1^. Minor shifts of anodic and cathodic peaks were observed for scan rate between 500 and 1000 mV s^−1,^ and anodic and cathodic currents for the films of **6**-EDOT here displayed an excellent linear dependence on scan rate. The gradients of current-scan rate plots for films prepared with the ratio of 1:5 (1 mM:5 mM) were the steepest, reflecting relatively facile anion insertion/ejection rates.

Table 3 presents data from multiple scan rate studies on the films of **6**-EDOT formed of different ratios using CV. No significant difference in Γ is seen, but slightly higher *i_p_* loss values were displayed by the films **6**-EDOT films compared to those of **2**-EDOT (Γ = 1.54–1.85 × 10^−9^ mol cm^−2^ and *i_p_* loss < 2.50%). Overall, the ΔE_p_ values increased with scan rate and those films made in ratio 1:1 (1 mM:1 mM) displayed the lowest ΔE_p_ values, implying high charge transport rates. Electropolymer films made in ratio 1:5 (1 mM:5 mM) showed the largest degree of variation in E_1/2_ values with scan rate, reflecting the least stability. Overall, the higher FWHM_ox_ values indicated a lower oxidation rate, and FWHM values of the EDOT films made in a ratio of 1:1 (1 mM:1 mM) were close to the theoretical value for n = 1 (90 mV/n).

SI 4(B) displays a Laviron plot of ΔE_p_ vs. log υ for the films of **6**-EDOT in a ratio of 1:5 (1 mM:5 mM) while Table 2 presents transfer coefficient and rate constant data (α and *k_s_*). **2**-pyrrole films resulted in *k_s_* values of 1.91–2.43 s^−1^ while films **6**-EDOT of 1:2.5 (1 mM:2.5 mM) showed the highest D_CT_^½^C of 2.1 × 10^−9^ mol cm^−2^ s^−1/2^ (product of the concentration of species and the charge transfer diffusion coefficient) with *k_s_* values of 1.04–1.06 s^−1^.

**Figure 4:**
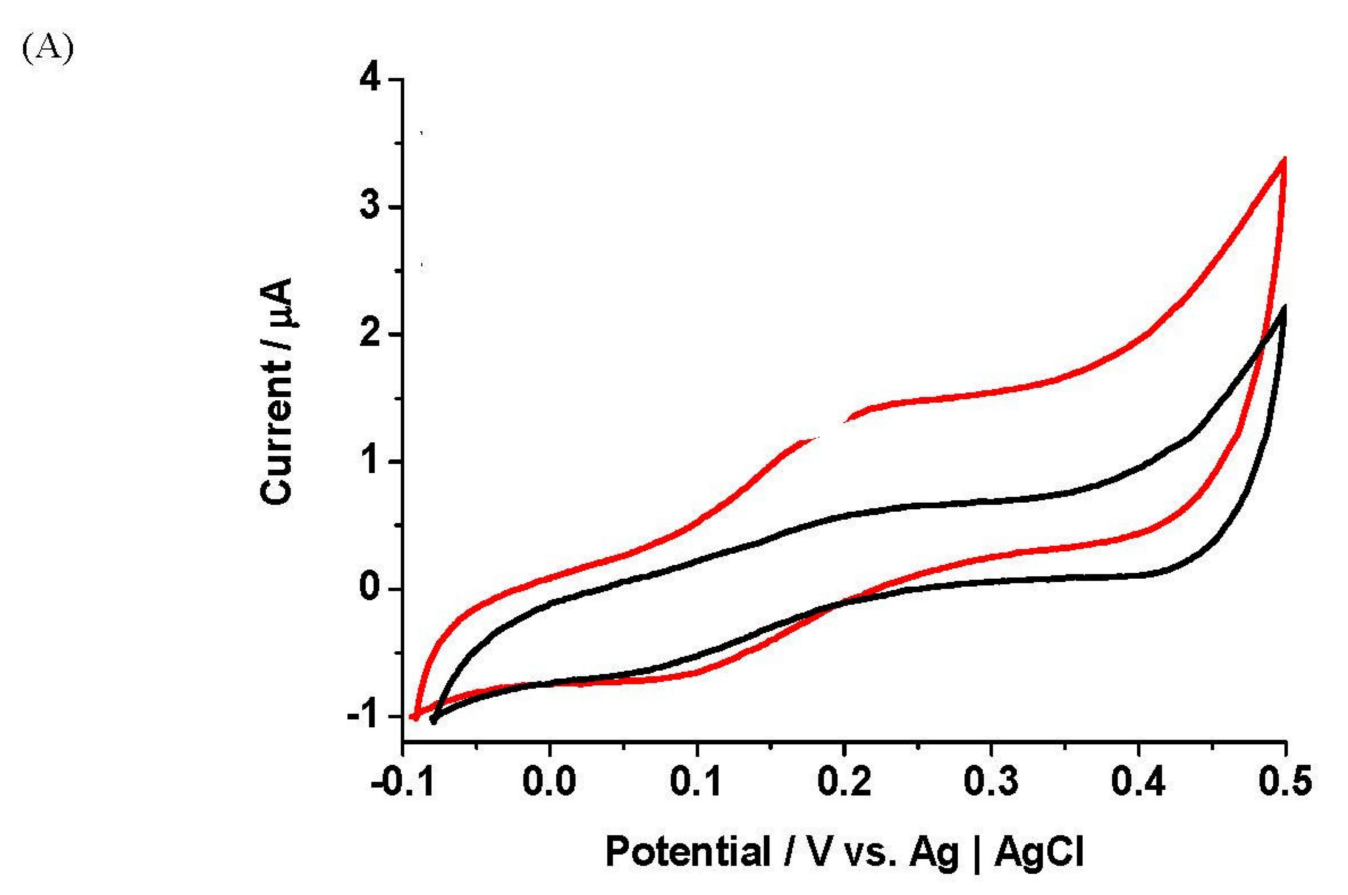

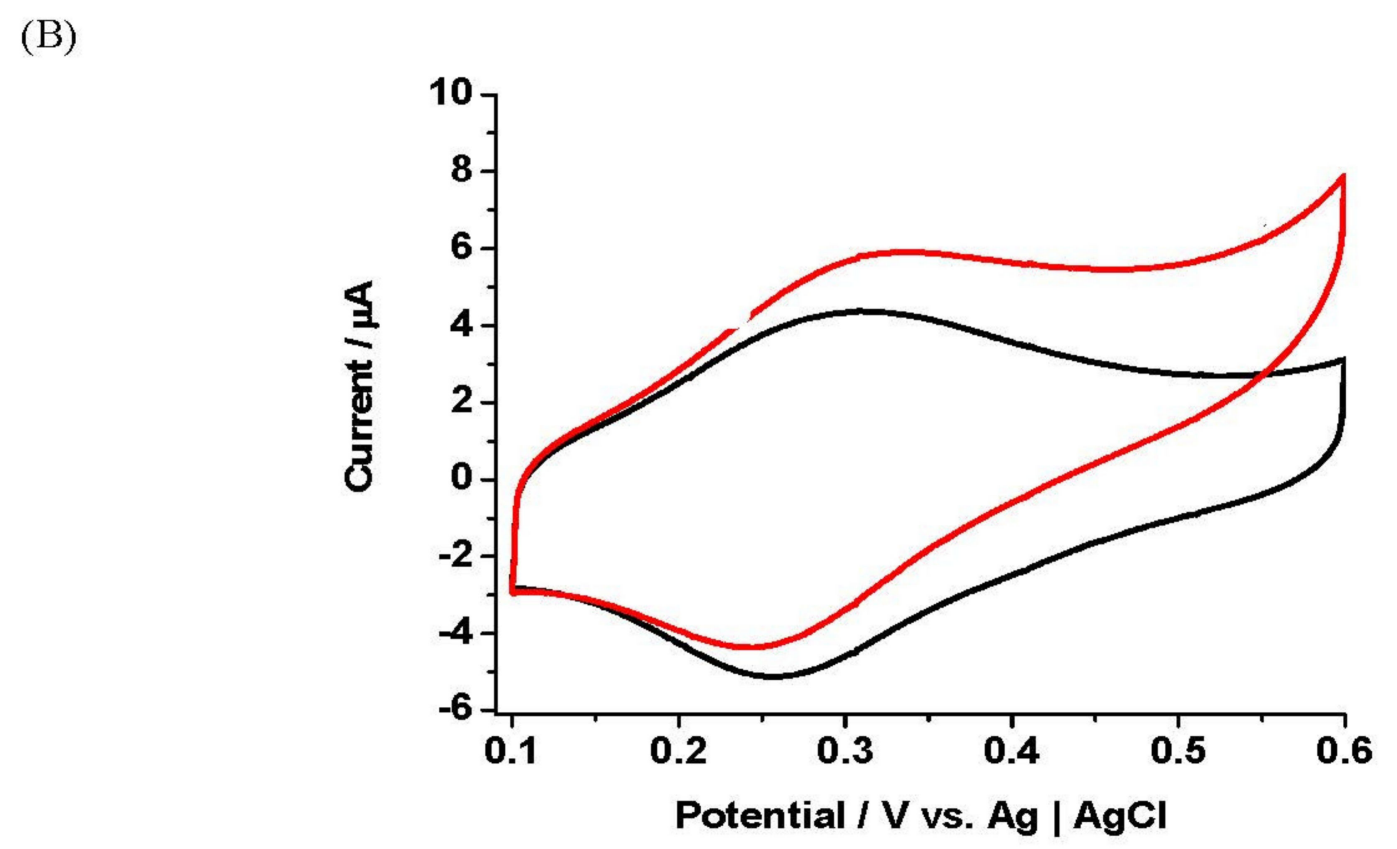
(**A**) Cyclic voltammetry responses of the films of 2-pyrrole in ratio of 1:1 (1 mM:1 mM) and GlutOx electrode towards addition of l-glutamate (10 mM) (red curve) Solution: PBS pH 7.2 (10 mM)-KCl (10 mM) (black curve). Potential sweep, −0.10–+0.50 V vs. Ag|AgCl, scan rate 0.5 mV s^−1^. Inset: Cyclic voltammetry response of the films and GlutOx at scan rate of 1 mV s^−1^. (**B**) Cyclic voltammetry responses of the films of 6-EDOT in ratio of 1:1 (1 mM:1 mM) and GlutOx electrode towards addition of l-glutamate (10 mM) (red curve) Solution: PBS pH 7.2 (10 mM)-KCl (10 mM) (black curve). Potential sweep, 0–+0.60 V vs. Ag|AgCl, scan rate 0.5 mV s^−1^. Inset: cyclic voltammetry response of the films and GlutOx at scan rate of 1 mV s^−1^.

Following the electrochemical film formation study, the mediated electrochemical detection of l-glutamate with films of PPy and EDOT modified by Fc derivatives was demonstrated. GlutOx was immobilised onto glassy carbon electrodes with underlying electroactive polymers according to the procedure in 2.3 above, resulting in a GCE/polymer film/enzyme layer. After overnight storage for equilibration, their redox stability profile and electrocatalytic behaviour towards l-glutamate was then evaluated using CV and chronoamperometry (CA). Only experiments employing the former technique at very low scan rates (0.5 mV s^−1^ and 1 mV s^−1^) showed positive results. Figure 4A shows electrocatalytic responses of films of **2**-pyrrole of 1:1 (1 mM:1 mM) enzyme electrodes with immobilised GlutOx showed sensitivities of 0.63 µA towards l-glutamate at very low scan rates of 0.5 mV s^−1^ and 1 mV s^−1^ (inset). Increased oxidation current, indicating a catalytic effect, and diminished reduction current were observed when l-glutamate (10 mM) was introduced. The anodic current response increased at +0.30 V, while the cathodic current diminished at +0.20 V when the substrates were added. The study was complete with detailed control experiments to verify the direct association of the generated catalytic response towards l-glutamate. Figure 4B presents the electrocatalytic responses of the films of **6**-EDOT of 1:1 (1 mM:1 mM) with immobilised GlutOx onto GCE surfaces towards l-glutamate. The current responses towards substrate (10 mM) increased and diminished at +0.40 V and +0.25 V, respectively. The modified GlutOx electrodes here showed noticeably lesser intensity in electrocatalytic responses (2.24 µA) to l-glutamate, compared to those generated by **6** in the buffer solution (8.3 µA in Figure 1B).

**Scheme 3:**
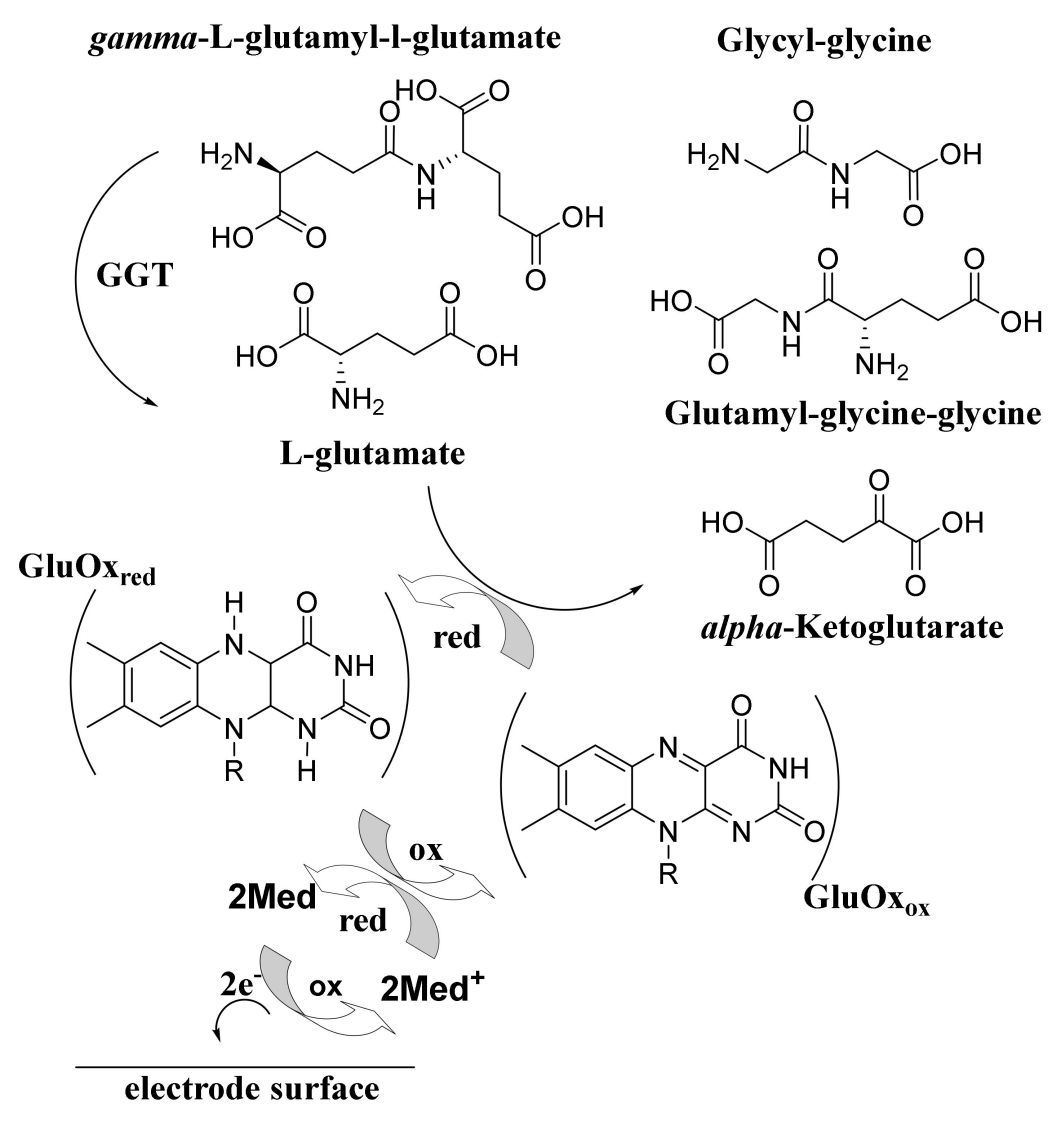
Proposed mediated electrochemical detection of GTP using GlutOx.

Extension of the methodology to GTP enzyme assay involved an examination of hydrogen peroxide electrooxidation at a Pt electrode as confirmation of unmediated GTP detection in the presence of co-substrates (Scheme 3).

**Figure 5:**
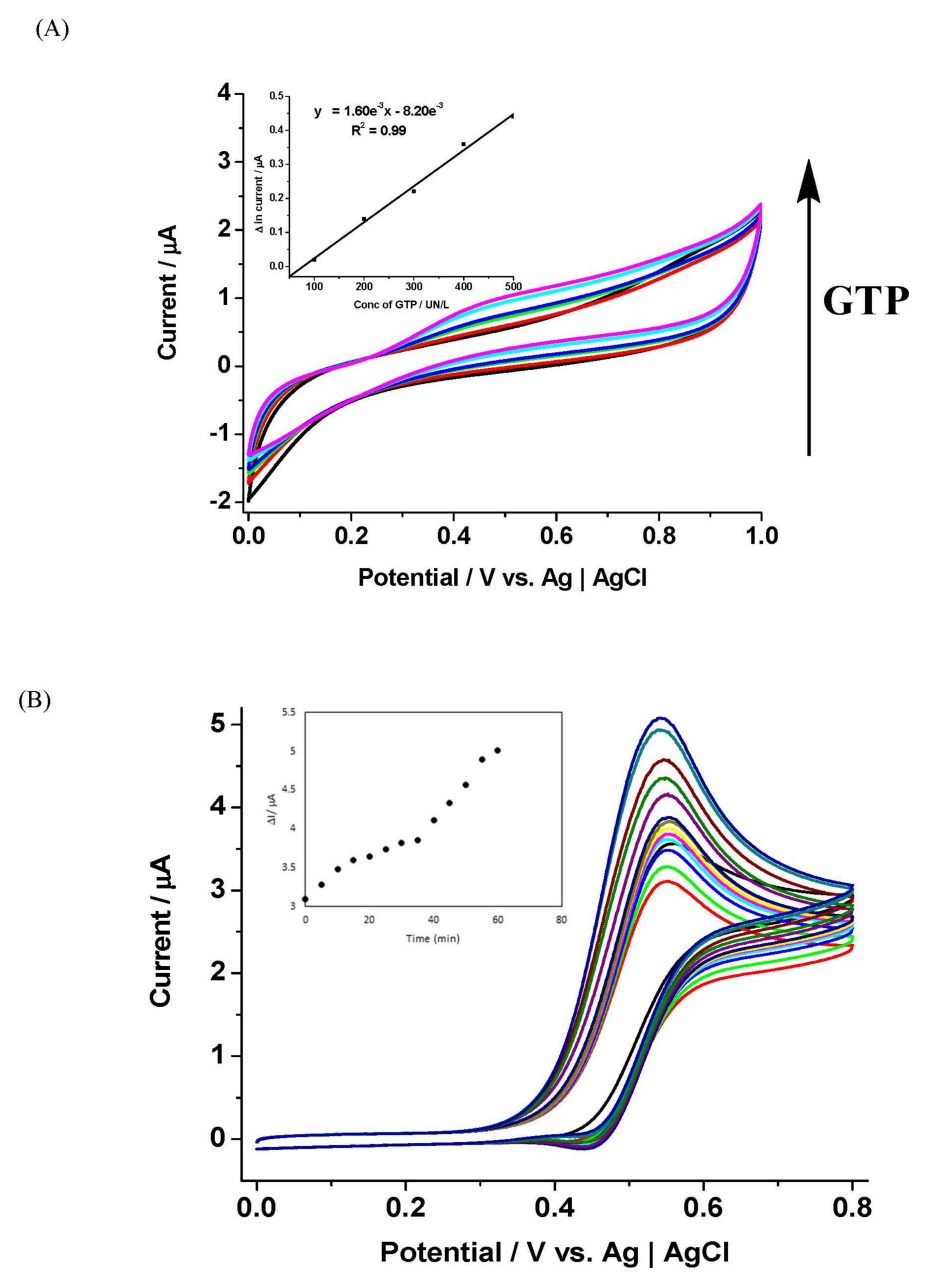
(**A**) Cyclic voltammetry responses of unmediated electrochemical detection towards successive additions of GTP (100–500 U/L) at a GlutOx-modified platinum working electrode. Solution: PBS pH 7.2 (10 mM)-KCl (10 mM) (black curve). Potential sweep, 0–+1.0 V vs. Ag|AgCl, scan rate 10 mV s^−1^. Inset: Calibration data showing a plot of the catalytic current versus GTP concentration. (**B**) Cyclic voltammetry responses of 1-hydroxy-2-[2-(thiophen-3-yl)-ethylamino]ethylferrocene (6) (1 mM) towards addition of GTP (600 U/L). Solution: PBS pH 7.2 (10 mM)-KCl (10 mM), γ-l-glutamyl-l-glutamate (10 mM) and glycyl-glycine (200 mM). Potential sweep, 0–+0.80 V vs. Ag|AgCl, scan rate 10 mV s^−1^. The experiment was conducted by running the mediator CV in substrate solution, followed by addition of GTP and a 5 min incubation. CVs were then run every 5 min for the next 60 min. Inset: plot of catalytic current (I_p,ox_) vs. time for the 6-GlutOx electrode response to high activity GTP.

Figure 5A shows unmediated electrochemical detection of GTP (100–500 U/L) with γ-l-glutamyl-l-glutamate (10 mM) and glycyl-glycine (200 mM) as the substrates at a GlutOx-modified platinum working electrode. The transfer of γ-glutamyl moiety from γ-l-glutamyl-l-glutamate to glycyl-glycine is catalysed by GTP at the solution-enzyme electrode interface. l-glutamate liberated at this interface is oxidised to *α*-ketoglutarate by GlutOx_ox_. The oxidation of GlutOx_red_ by O_2_ generates H_2_O_2_ in the protein layer of the electrode. Peroxide is then detected by electrooxidation at the platinum electrode surface. Increases in the oxidation current with GTP concentration were observed with the electrode potential set at +0.55 V and indicated a catalytic effect. A calibration graph (inset) shows the background-subtracted current response of the GlutOx electrode towards GTP concentration. Unmediated electrochemical detection of GTP at a GlutOx electrode was demonstrated in this work, with investigations performed at much lower enzyme activity than previously reported, i.e., 5.4 U/L. In control studies with platinum electrodes not supporting a GlutOx layer, there was no significant change in current for the transfer of γ-glutamyl moiety from γ-l-glutamyl-l-glutamate in the presence of GTP. This result indicates that the GlutOx electrode was responsive to l-glutamate generation from the preceding GTP reaction.

Morimoto et al. reported unmediated electrochemical detection of GTP using γ-l-glutamyl-l-glutamate and glycyl-glycine, which was successfully reproduced using our GlutOx biosensor (Morimoto et al., 2007). In this prior work, a linear relationship between the response curve slope and GTP activity of 35–659 U/L using HA was achieved; the biosensor reported here had an analytical limit of detection for GTP activity of as low as ~5 U/L. The GTP study in this work employed other electrochemical techniques such as CV and CA for the determined enzyme activities (100–500 U/L). Linear correlations were observed between current response gradients and GTP activities.

Having established the unmediated electrochemical detection of GTP using γ-l-glutamyl-l-glutamate as its substrate, the next stage was to incorporate a redox mediator into the biosensor system for electron-mediated detection of the GTP enzyme. Preliminary data for the redox mediator, 1-hydroxy-2-[2-(thiophen-3-yl)-ethylamino]ethylferrocene (**6**) is reported here. Further studies with the **6**-GlutOx electrode were conducted with higher GTP activity solutions (600 U/L), and the response measured with reaction time over an extended period of 1 hour. Figure 5B shows the cyclic voltammetry behaviour of the **6**-GlutOx electrode to the presence of a high GTP enzyme activity level. The shapes of the voltammetric waves in these experiments are noticeably altered compared to the low concentration studies. The oxidation wave is now a main feature, which is found to increase with enzyme-reaction time. The reduction wave, by contrast, vanishes. There was also a shift in the E_p,ox_ potential in the cathodic direction. The plot of catalytic current, I_p,ox_ with time for the **6**-GlutOx electrode response to high GTP activity showed a plateau followed by a rise in current at longer enzyme reaction times following the addition of GTP (inset). The mediator I_p,ox_ was 3.6 µA (GTP 0 U/L) for this electrode, compared to I_p,ox_ 3.1 µA after the introduction of GTP (600 U/L). The electrode was responsive to GTP activity, and the catalytic current increased with reaction time for the next 60 min (rate of response 27.5 nA/min). This data indicates that the **6**-GlutOx electrode was sensitive to GTP and suggests the mediated-electrode system could detect high enzyme activity after a few minutes from the start of the enzyme reaction.

When the electrode response to GTP concentration was investigated using cyclic voltammetry for the **6**-GlutOx electrode over the range 0–500 U/L (SI 5), the electrode was responsive to GTP at low concentration and became unresponsive at concentrations above approximately 200 U/L, where a limiting catalytic current was observed (1.95 µA). At low concentration, the current-concentration response of the electrode was approximately linear (R^2^ = 0.97), with a sensitivity of the order of 5 nA/UL^−1^ (<100 U/L). GTP was incubated with γ-l-glutamyl-l-glutamate and glycyl-glycine before adding the mixture into the cell with **6**-GlutOx for analysis. Hence, an incubation process ranging from 5 min to up to 5 h was carried out on combinations of γ-l-glutamyl-l-glutamate (10 mM), glycyl-glycine (200 mM) and GTP (100 U/L) before analysing with **6**-GlutOx. No significant difference in the anodic current responses was observed (data not shown here). This verified that an incubation time of ≤ 6 min was adequate to generate l-glutamate from γ-l-glutamyl-l-glutamate by GTP. It was suspected that there might be interferences between the enzymes, GlutOx and GTP, and substrates, γ-l-glutamyl-l-glutamate, glycyl-glycine and l-glutamate. Furthermore, the incubation of different combinations of the enzymes and substrates with Fc derivative **6** was implemented. When γ-l-glutamyl-l-glutamate and GTP were introduced without glycyl-glycine as glutamyl acceptors, anodic current responses increased at +0.35 V (data not shown). This response might be due to redox reactions between γ-l-glutamyl-l-glutamate, GTP and Fc mediator **6** and requires further investigation.

## 4. Conclusions

Synthesis of 3-(1*H*-pyrrol-1-yl)propanamidoferrocene (**2**) and 1-hydroxy-2-[2-(thiophen-3-yl)-ethylamino]ethylferrocene (**6**) followed by characterisation using TLC, FTIR, ^1^H NMR, ^13^C NMR and elemental analysis resulted in two new candidates for redox-active polymer deposition with the aid of pyrrole and EDOT monomers on electrode surfaces followed by a study into their redox film behaviour. Mediation capabilities towards l-glutamate were tested using electrochemical techniques such as CV, chronoamperometry and hydrodynamic amperometry. The data obtained provided valuable insights into understanding film growth and electrochemical parameters via co-polymerisation with solution-phase redox mediation of glutamate in addition to the first-time extension to the electrochemical assay of liver enzyme GTP.

These Fc derivatives displayed very good solubility in aqueous media with low E_1/2_ values (0.18 and 0.26 V for (**2**) and (**6**), respectively), enabling selectivity in the presence of expected interferences in real biological samples. Mediated voltammetric glutamate investigations at a glutamate oxidase electrode measurement resulted in steady-state current responses over the range 1–4 mM with sensitivities of 0.86 and 1.28 μA/mM for (**2**) and (**6**), respectively (solution studies). Immobilised ferrocene species exhibited a viable response by CV but with lower sensitivities, pointing towards a possible limitation of redox sites and/or sluggish rates of enzyme reoxidation due to size/charge effects at the enzyme-modified electrode.

Data from the first-time report of mediated electrochemical detection of liver biomarker GTP led to the initial conclusion that a preincubation process consisting of GTP, γ-l-glutamyl-l-glutamate and glycyl-glycine was essential to allow l-glutamate to be produced in sufficient quantities, and hence electrocatalytic responses were generated and detected by the enzyme electrode. The ability to address quantitation of such a challenging biomarker is highly promising, and electrochemical data achieved here provide confidence that the solution phase and immobilised mediation capabilities of these compounds will lead to deployment in biosensing applications, e.g., glutamate neurochemical studies and or liver panel diagnostic arrays. Therefore, the work establishes the groundwork for the examination of a range of pyrrole/thiophene ferrocene derivatives in applications such as those presented here, with exciting opportunity areas in liver-panel-multiplexed electrochemical enzyme assays.

## Figures and Tables

**Table 1:** Data from variable scan rate study on the films of 3-(1*H*-pyrrol-1-yl)propanamidoferrocene (**2**) and pyrrole using CV.

Ratio of Fc:pyrrole (mM)	10^9^ Γ (mol cm^−2^)	*i*_pa_ Loss (%)	*i*_pc_ Loss (%)	Scan Rate (mV s^−1^)	ΔE_p_ (mV)	E_1/2_ (mV)	FWHM_ox_ (mV)	FWHM_red_ (mV)
**1:1** **(1 mM:1 mM)**	0.33	4.66	5.15	1	86	267	208	200
				5	62	276		
				10	95	279		
				20	128	282		
				50	189	284		
				100	248	276		
				200	399	277		
**1:2.5** (1 **mM:2.5 mM)**	0.14	3.73	2.30	1	104	217	224	168
				5	135	230		
				10	187	236		
				20	232	235		
				50	325	228		
				100	371	225		
				200	407	224		
**1:5** **(1 mM:5 mM)**	0.47	5.11	4.28	1	111	206	204	158
				5	171	206		
				10	246	212		
				20	300	200		
				50	349	205		
				100	359	208		
				200	369	207		

**Table 2:** Data on D_CT_^½^C, α and *k_s_* of the films of (3-(1*H*-pyrrol-1-yl)propanamido) ferrocene (**2**) and pyrrole and 1-hydroxy-2-[2-(thiophen-3-yl)-ethylamino]ethylferrocene (**6**) with EDOT.

*(3-(1H-pyrrol-1-yl)propanamido) ferrocene (2)—pyrrole*			
**Ratio of Fc:pyrrole** (**mM**)	**10^−9^ D_CT_^½^C** (**mol cm^−2^ s^−1/2^**)	**α**	***k_s_*** (**s^−1^**)
1:1 (1 mM:1 mM)	0.53	0.22	1.19
1:2.5 (1 mM:2.5 mM)	0.26	0.48	2.41
1:5 (1 mM:5 mM)	0.53	0.42	1.54
*1-hydroxy-2-[2-(thiophen-3-yl)-ethylamino]ethylferrocene (6)—EDOT*			
**Ratio of Fc:EDOT** (**mM**)	**10^−9^ D_CT_^½^C** (**mol cm^−2^ s^−1/2^**)	**α**	***k_s_*** (**s^−1^**)
1:1 (1 mM:1 mM)	0.42	0.80	1.04
1:2.5 (1 mM:2.5 mM)	2.10	0.78	1.09
1:5 (1 mM:5 mM)	0.53	0.53	1.46

Mediated electrochemical detection of l-glutamate.

**Table 3:** Data from the multiple scan rate study on the films of 1-hydroxy-2-[2-(thiophen-3-yl)-ethylamino]ethylferrocene (**6**) and EDOT using CV (FWHPH full width at half peak maximum).

Ratio of Fc:EDOT (mM)	10^9^ Γ (mol cm^−2^)	*i*_pa_ Loss (%)	*i*_pc_ Loss (%)	Scan Rate (mV s^−1^)	ΔE_p_ (mV)	E_1/2_ (mV)	FWHM_ox_ (mV)	FWHM_red_ (mV)
1:1 (1 mM:1 mM)	1.72	5.02	1.75	1	70	315	166	90
				5	75	314		
				10	70	316		
				20	71	316		
				50	74	314		
				100	84	318		
				200	78	319		
				500	95	322		
				800	107	331		
				1000	112	332		
1:2.5 (1 mM:2.5 mM)	1.60	4.83	3.76	1	30	336	210	148
				5	62	356		
				10	66	358		
				20	71	362		
				50	78	361		
				100	89	360		
				200	106	355		
				500	119	352		
				800	121	351		
				1000	135	352		
1:5 (1 mM:5 mM)	1.15	5.05	1.00	1	44	322	170	136
				5	53	326		
				10	62	326		
				20	72	325		
				50	62	322		
				100	75	328		
				200	79	334		
				500	100	338		
				800	104	347		
				1000	110	343		

## Data Availability

Not applicable.
